# Depression and Coping Strategies Among Palliative Care Patients in a Tertiary Hospital in Karachi, Pakistan

**DOI:** 10.1177/26892820251394288

**Published:** 2025-11-17

**Authors:** Hina Ali, Ismat Jabeen, Nawazish Zehra

**Affiliations:** ^1^ Department of Family Medicine, Aga Khan University, Karachi, Pakistan.; ^2^ Department of Oncology, Aga Khan University Hospital, Karachi, Pakistan.

**Keywords:** coping, depression, Pakistan, palliative care, psychological distress

## Abstract

**Background:**

Depression is a common yet under-recognized condition among palliative care patients, often affecting their psychological well-being and quality of life. Coping strategies used by these patients may influence their mental health outcomes and overall adaptation to illness.

**Objective:**

To assess the presence of depression and identify coping strategies among palliative care patients attending a tertiary teaching hospital in Karachi, Pakistan.

**Setting/Subjects:**

This cross-sectional study was conducted at Aga Khan University Hospital, Karachi, Pakistan, over six months (June to December 2023). A total of 81 adult patients receiving palliative care for at least one month were enrolled in the study. Depression was assessed using the Patient Health Questionnaire-9, while coping strategies were evaluated using the Brief COPE Inventory. Descriptive and inferential statistics were applied using SPSS v. 21.

**Results:**

Depression was present in majority of participants, with 30% showing minimal, 18% mild, 19% moderate, 22% moderately severe, and 11% severe symptoms. The coping styles used were problem-focused (2.65 ± 0.65), emotion-focused (2.18 ± 0.32), and avoidant coping (1.74 ± 0.34). Female participants were more likely to employ emotion-focused coping compared to male participants (*p* = 0.012). A weak inverse correlation was observed between education level and depression severity (*r* = −0.213, *p* = 0.056).

**Conclusion:**

Depression is highly prevalent among palliative care patients in this context. Most patients adopt adaptive coping strategies, particularly emotional support and religious coping. These findings highlight the need for integrated psychological care as a routine component of palliative treatment.

## Key Message

Depression is a significant concern in palliative care, impacting patients’ quality of life and their ability to cope with their illness. Recognizing and addressing depression in palliative care settings is essential for providing holistic care and improving patient outcomes.

## Introduction

Pakistan is a developing country with a growing population and an under-resourced health care system. It faces a dual burden of communicable and noncommunicable diseases, with an estimated 3.9 million deaths expected between the ages of 30 and 69 due to these illnesses by 2025.^1,2^ As the prevalence of chronic illnesses rises, the need for comprehensive palliative care services becomes increasingly critical.

Palliative care aims to improve the quality of life of patients and their families facing life-threatening illnesses by addressing physical, psychosocial, and spiritual suffering.^3^ Globally, palliative care is recognized as an essential component of health care. However, in Pakistan, the availability of such services remains limited. According to Lynch, Connor, and Clark, the ratio of palliative care services to population in Pakistan is alarmingly low, estimated at 1:90 million.^4^

Patients with serious, life-limiting illnesses often experience psychological distress, including symptoms ranging from sadness to major depressive disorder.^5,6^ Depression in palliative care settings is a significant concern, with reported global prevalence ranging from 7% to 58%.^6^ The World Health Organization (WHO) defines depression as a common mental disorder characterized by persistent sadness, loss of interest or pleasure, feelings of guilt or low self-worth, disturbed sleep or appetite, fatigue, and poor concentration.^5^ If left unaddressed, depression in these patients can lead to impaired immunity, decreased quality of life, poor treatment adherence, and even increased mortality.^5^

To manage psychological distress, patients often employ various coping mechanisms. Coping refers to the cognitive and behavioral efforts made to manage internal or external stressors that are perceived as overwhelming.^7^ Studies from different regions suggest that the nature of coping, whether adaptive (e.g., emotional support, acceptance, problem focused) or maladaptive (e.g., denial, self-blame), can significantly influence a patient’s psychological state and quality of life.^6^

For instance, a study from the United States involving incurable cancer patients found that emotional support and acceptance were associated with better mood outcomes, while denial and self-blame correlated negatively with mental well-being.^7^ Emotional support is defined as “an individual’s expression of sympathy, understanding, respect and encouragement to others through verbal and nonverbal behaviours.”^8^ Furthermore, acceptance coping is regarded as “a self-regulation strategy influenced by a welcoming and open attitude toward one’s own thoughts, emotions, or external factors.”^9^

A study from India found that religion and acceptance of the terminal illness were the most common coping strategies among palliative care patients, with notable gender differences. Females used more problem-focused coping, while males utilized emotional and avoidant strategies.^10^ Problem-focused coping represents “the active efforts to manage stressful situations and change a troubled individual-environment relationship to eliminate or alter the sources of stress via a person’s behaviour.”^11^ This means that individuals may take practical steps to reduce or fix the source of stress.

Despite increasing awareness of the importance of palliative care, there is limited research on depression and coping strategies in palliative care settings in Pakistan. As the burden of chronic and terminal illnesses continues to grow, understanding the psychosocial needs of these patients becomes essential for developing effective, culturally relevant care models.

This study was conducted to address this gap by assessing the prevalence of depression and identifying coping strategies among palliative care patients in a tertiary care hospital in Karachi. The findings aim to support clinicians and policymakers, so that they are aware that palliative care patients may develop depression and therefore include psychosocial interventions in their consultations with an aim to improve their quality of life.

### Ethical approval

This study was approved by the Ethics Review Committee of Aga Khan University Hospital (ERC reference number, 2023-6900-23732).

## Methodology

A prospective cross-sectional study was conducted in the Department of Family Medicine at Aga Khan University Hospital, Karachi. Participants were recruited from both inpatient and outpatient palliative care settings. Eligibility criteria included patients aged between 18 and 80 years who had been receiving palliative care services for at least one month and provided written informed consent.

The sample size was determined using the WHO sample size calculator.^12^ Similar research carried out in India was used as a reference study to calculate sample size. In this study, 70% of palliative care patients were reported to suffer from depression, and this parameter was used to calculate the sample size.^4^ A nonprobability sampling technique was employed for patient recruitment. Nonprobability sampling is a sampling method that utilizes nonrandom criteria including availability and accessibility, in order to answer a research question.^13^

Data were collected using a structured, predesigned proforma, which included variables such as age, gender, ethnicity, marital status, type of family, and comorbidities.

Depression was assessed using the Patient Health Questionnaire-9 (PHQ-9),^14^ while coping strategies were evaluated through the Brief COPE Inventory.^15^ To ensure participant confidentiality, all questionnaires were anonymized, and completed forms were securely stored in a locked cabinet within the department.

### Statistical analysis

Data were analyzed using SPSS version 21.0 (IBM, Armonk, NY). Descriptive statistics were used to summarize demographic characteristics, coping strategies, and depression severity. Chi-squared tests were applied to assess the association between categorical variables (gender, occupation, education level). Pearson correlation coefficients were calculated to examine the relationships between continuous variables, including coping strategies and depression scores. For multivariable analysis, a general linear model was employed to identify independent predictors of depression while adjusting for potential confounders such as age, gender, marital status, education level, comorbidities, and duration of illness. Additionally, graphs were utilized to visualize linear trends and relationships between coping strategies and relevant demographic or clinical variables.

## Results

A total of 81 participants were included in the study. The majority of participants were aged 70 and over (53.1%), followed by those aged 60–69 years (22.2%), 50–59 years (16.1%), 40–49 years (4.9%), and 30–39 years (3.7%). Males constituted 46.9% of the sample, while females made up 53.1%. Most participants were married (82%), whereas 14% were widowed and 4.2% were single. Regarding family structure, a large proportion (82%) belonged to combined families, while 18.7% lived in nuclear families. In terms of education, 37.2% were graduates, 23.4% had completed intermediate education, 22.2% had secondary education, 12.3% had primary education, and only 4.9% held a postgraduate degree. Occupationally, more than half of the participants were unemployed (53%), followed by homemakers (22.2%), office workers (19.7%), professionals (2.4%), and laborers (2.4%). The duration of terminal illness (including end-stage cardiovascular diseases, cancer, chronic respiratory diseases, kidney failure, and chronic liver disease) varied. Most patients (38.5%) experienced the illness for 5 years, whereas other durations seen were 1 year (20.9%), 2 years (13.5%), 3 years (18.5%), 4 years (3.7%), 6 years (1.2%), and 10 years (3.7%). The demographic information of the participants is provided in Table 1.

**Table 1.** Demographics

Variables	Categories	Frequency	Percentages
Age	30–39	3	3.7
	40–49	4	4.9
	50–59	13	16.1
	60–69	18	22.2
	70 and above	43	53.1
Gender	Male	38	46.9
	Female	43	53.1
Marital status	Married	67	82
	Single	02	4
	Widowed	12	14
Type of family	Nuclear	15	18
	Combined	66	82
Education level	Primary	10	12.3
	Secondary	18	22.2
	Intermediate	19	23.4
	Graduate	30	37.2
	Postgraduate	04	4.9
Occupation	Homemaker	18	22.2
	Labor	02	2.4
	Office Worker	16	19.7
	Professional	02	2.4
	Unemployed	43	53.3
Duration of illness (years)	1	17	20.9
	2	11	13.5
	3	15	18.5
	4	03	3.7
	5	31	38.5
	6	01	1.2
	10	03	3.7

### Depression scale PHQ-9

**FIG. 1.**
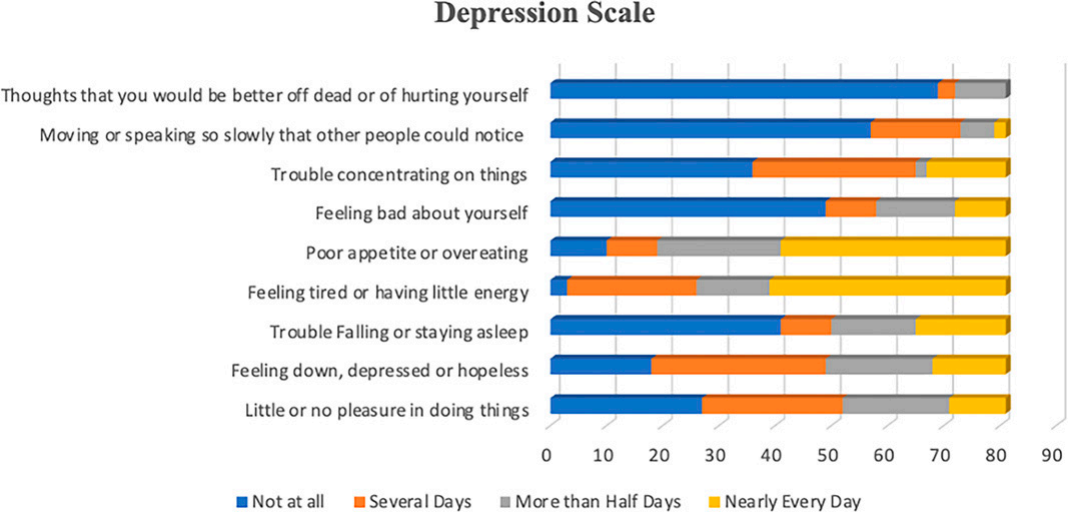
Depression Scale.

The analysis of depressive symptoms using a standardized depression scale, PHQ 9, revealed that several symptoms were reported with high frequency among participants as shown in Figure 1. Analysis of responses revealed varying levels of depressive symptoms among participants. About one-third (33.3%) reported having little or no pleasure in doing things, while 30.9% experienced this several days of the week, almost 12.3% experienced this nearly every day. Feelings of being down, depressed, or hopeless were reported several days by 38.3% of participants and nearly every day by 16.0%.

More than half (50.6%) had no trouble falling or staying asleep, although 19.8% reported sleep difficulties nearly every day. In contrast, more than half of the participants (51.9%) reported feeling tired or having little energy nearly every day. Poor appetite or overeating was common, with 49.4% experiencing it nearly every day.

While 60.5% patients did not feel bad about themselves at all, 17.3% reported this feeling more than half the days or nearly every day. Difficulties in concentration were absent in 44.4% of participants, but 11.1% reported them nearly every day. Psychomotor changes were largely absent, with 70.4% reporting no such difficulties.

Importantly, suicidal ideation was absent in the majority (85.2%), though 3.7% reported it on several days and 11.1% reported it more than half the days over the last two weeks.

### Severity of depression

**FIG. 2.**
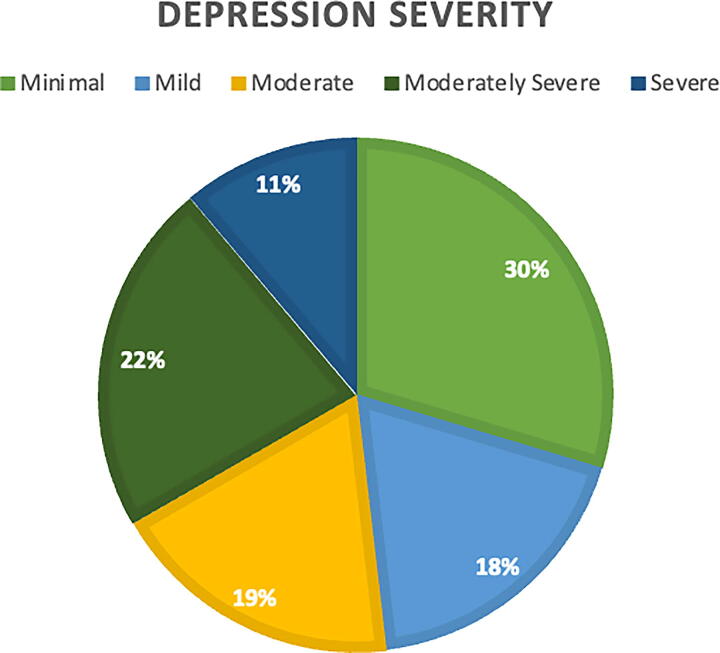
Depression severity.

Based on the categorization of depression severity scores among the 81 participants, the findings indicate that a substantial proportion of individuals are experiencing clinically significant depressive symptoms. Approximately 30% of participants fell within the minimal depression range (scores 1–4), suggesting relatively low levels of depressive symptoms. Mild depression (scores 5–9) was observed in 18.0% of participants, while moderate depression (scores 10–14) was present in 19% of the sample. Notably, 22.0% of participants were categorized as having moderately severe depression (scores 15–19), and 11% were identified with severe depression (scores 20–27). Overall, more than half of the participants (52%) exhibited moderate to severe depressive symptoms, indicating a considerable mental health burden within the population studied. Figure 2 demonstrates the severity of depression among the participants.

### Coping strategies (Coping Inventory)

**FIG. 3.**
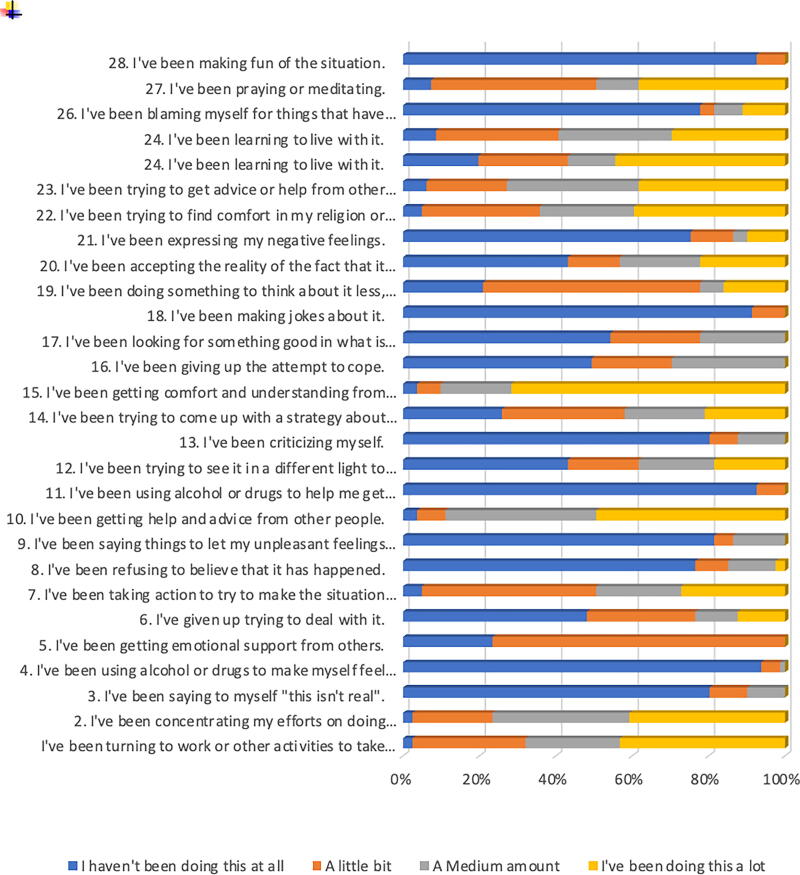
Coping strategies.

The results indicate that most participants predominantly used adaptive coping strategies such as seeking emotional support, problem-solving, and religious coping. High frequencies were reported for behaviors such as seeking advice, comfort, and engaging in prayer or meditation. Emotion-focused strategies such as acceptance and positive reframing were moderately used. In contrast, avoidant and maladaptive strategies including substance use, denial, and humor were rarely endorsed. Self-blame and emotional venting were also infrequent. Overall, the participants demonstrated a constructive approach to coping with stress, favoring supportive and action-oriented methods over avoidance. Figure 3 illustrates the range of coping strategies used by patients.

### Univariate analysis

Univariate analysis was conducted to evaluate the association between depression scores and various demographic and clinical characteristics among study participants. In addition, depression score was treated as a continuous outcome variable. Mean depression scores were compared across categorical variables (e.g., gender, marital status, education, occupation) using *t* tests/analysis of variance, while correlations/regression were used for continuous predictors (age, duration of illness). The results revealed no statistically significant association between depression scores and duration of illness (*p* = 0.416), marital status (*p* = 0.548), or presence of comorbid conditions (*p* = 0.535). Similarly, age (*p* = 0.602) and occupation (*p* = 0.886) were not significantly associated with depression scores. A borderline significant association was noted for educational level, which showed a modest effect on depression scores (*p* = 0.056), suggesting that individuals with different levels of education may experience varying degrees of depressive symptoms. Gender also approached statistical significance (0.065), indicating a potential trend toward gender differences in depression levels, although this did not reach the conventional threshold for significance. Overall, the analysis shows that most of the examined variables did not significantly impact depression scores in this sample.

### Multivariable analysis

In the multivariable analysis using a univariate general linear model, several sociodemographic and clinical variables were evaluated simultaneously to assess their collective association with depression scores. The overall model was not statistically significant (*p* = 0.592), indicating that the included variables explained only a small proportion of the variance in depression scores (*R*^2^ = 0.059; adjusted *R*^2^ = −0.017). None of the individual predictors showed a statistically significant effect. Gender approached significance (*p* = 0.091), suggesting a potential trend, but this did not meet the conventional threshold for significance. Other variables, including marital status (*p* = 0.739), age (*p* = 0.871), occupation (*p* = 0.891), presence of comorbidities (*p* = 0.681), and duration of illness (*p* = 0.448), were not significantly associated with depression scores.

### Correlations and associations

Correlation and association analyses were performed between all key study variables to explore potential relationships. While most variables did not demonstrate statistically significant relationships, a few showed meaningful correlations and linear associations. These significant findings are detailed below and provide insight into the interplay between coping strategies, demographic factors, and psychological outcomes.

### Correlation between depression and education level

The Pearson correlation analysis revealed a negative correlation between depression scores and educational level (*r* = −0.213, *p* = 0.056) as shown in Table 2. This indicates a weak inverse relationship, suggesting that individuals with higher educational levels tend to report slightly lower depression scores. However, the association did not reach statistical significance at the conventional alpha level of 0.05, as the *p* value is marginally above the threshold (*p* = 0.056). Therefore, while there appears to be a trend suggesting that higher education may be associated with lower levels of depressive symptoms.

**Table 2.** Correlation Between Depression and Education Level

	Depression score	Educational level
Depression score		
Pearson correlation	1	−0.213
Sig. (two-tailed)		0.056
* N*	81	81
Educational level		
Pearson Correlation	−0.213	1
Sig. (two-tailed)	0.056	
* N*	81	81

### Correlation between emotion-focused coping and gender

The Pearson correlation analysis demonstrated a statistically significant positive correlation between emotion-focused coping scores and gender (*r* = 0.277, *p* = 0.012) as indicated in Table 3. This indicates a moderate association, suggesting that gender is significantly related to the use of emotion-focused coping strategies. Given that gender is typically coded as a binary variable (e.g., 1 = male, 2 = female), the positive correlation implies that female participants are more likely to utilize emotion-focused coping mechanisms compared to their male counterparts. This relationship is statistically significant at the 0.05 level, indicating that the observed association is unlikely due to chance.

**Table 3.** Correlations Between Emotion-Focused Coping and Gender

	Emotional_focused_coping	Gender
Emotional_focused_coping_avg		
Pearson correlation	1	0.277*
Sig. (two-tailed)		0.012
* N*	81	81
Gender		
Pearson correlation	0.277*	1
Sig. (two-tailed)	0.012	
* N*	81	81

*Correlation is significant at the 0.05 level (two-tailed).

### Association between emotion-focused coping and gender

**FIG. 4.**
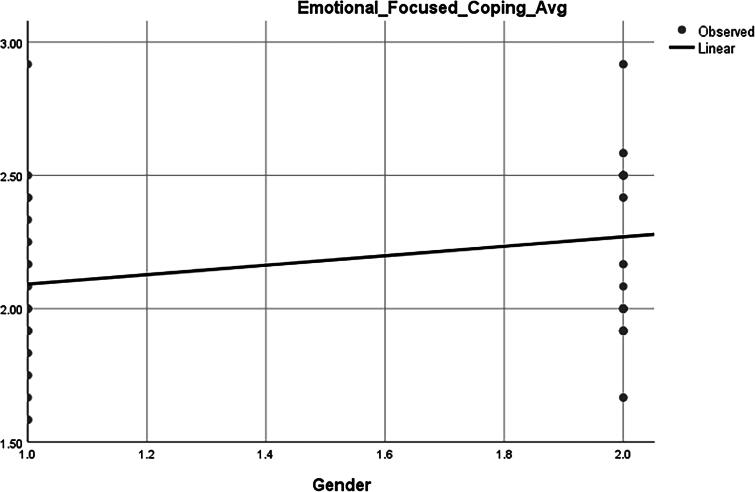
Gender.

The plot (Fig. 4) visually supports the earlier correlation analysis, showing that females (gender = 2) tend to report slightly higher emotion-focused coping scores compared to males (gender = 1). While the slope is not steep, it reflects a positive association between being female and greater use of emotion-focused coping mechanisms. This aligns with the statistically significant Pearson correlation (*r* = 0.277, *p* = 0.012), suggesting that gender plays a modest but meaningful role in influencing emotional coping styles.

### Association between avoidant coping and occupation

**FIG. 5.**
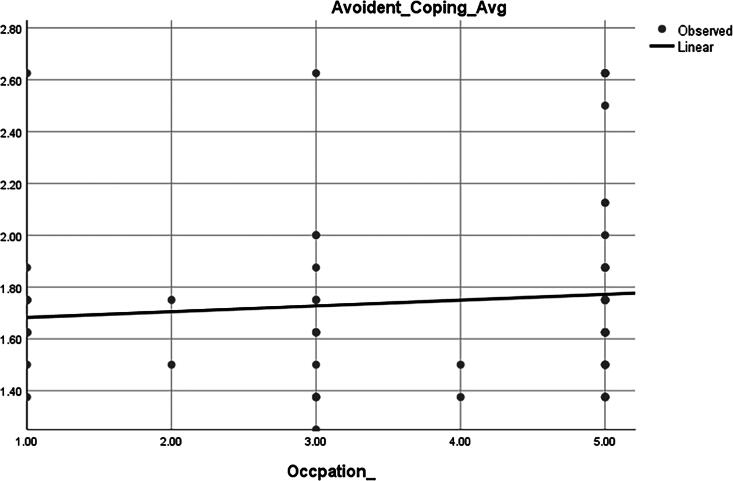
Occupation.

The plot (Fig. 5) suggests a minor positive association between occupational status and the tendency to use avoidant coping strategies. However, the weak slope implies that occupation may not be a strong predictor of avoidant coping in this sample and should be interpreted cautiously, ideally with supporting statistical significance from a correlation or regression test.

## Discussion

This study examined the prevalence of depression and the coping strategies used by palliative care patients at a tertiary hospital in Karachi, Pakistan. Moderate to severe depression was found in approximately 52% of participants. These findings reflect a significant psychological burden among palliative care patients in a local context and align with global estimates, which report prevalence rates ranging from 7% to 58%.^15,16^

Common depressive symptoms identified included anhedonia, fatigue, appetite changes, and feelings of hopelessness. Although suicidal ideation was infrequently reported, its presence in any capacity highlights the need for proactive mental health screening and support within palliative care services.^17,18^

Most participants reported using adaptive coping mechanisms, primarily emotional support, religious coping, and acceptance. These findings are consistent with previous studies from both high-income and regional settings, which suggest that spiritual and emotional coping are prevalent among patients with terminal illness.^18,19^ The relatively low use of avoidant strategies such as substance use and denial further suggests a constructive coping approach among this population.

Gender differences were noted, with female patients more likely to employ emotion-focused coping, a finding consistent with other literature indicating that women are more inclined to seek social support and use emotional expression as a coping strategy.^20,21^

An inverse, though statistically borderline, correlation between educational level and depression was observed. Higher education may contribute to better health literacy and psychological resilience, as previously documented in the literature,^22,23^ although this relationship warrants further investigation in larger samples.

Importantly, no significant associations were found between depression and variables such as age, marital status, duration of illness, or comorbidities. This suggests that psychological distress in palliative care may be influenced more by individual psychological and contextual factors than by sociodemographic characteristics alone.^24–26^

Strengths of the study include the use of validated instruments (PHQ-9 and Brief COPE), a structured data collection approach, and appropriate statistical analysis. However, several limitations must be acknowledged:

• The sample size was relatively small (*n* = 81), which may limit generalizability.
• Nonprobability sampling could introduce selection bias.
• The cross-sectional design precludes causal inferences.
• Findings are based on self-reported data, which may be subject to reporting bias.

## Conclusion

Despite the limitations, this study contributes valuable insights into the psychosocial aspects of palliative care in Pakistan. It highlights the urgent need to integrate psychological screening and support services into routine palliative care protocols. Interventions such as counseling, spiritual support, and, where appropriate, pharmacological treatment may improve the quality of life for patients facing life-limiting illnesses.

Future research should explore these relationships longitudinally and across a more diverse patient population to better understand the causes of depression among the palliative care patients and evolution of coping and psychological distress over time.

## Ethical Approval

This study was approved by the Ethics Review Committee, Aga Khan University (ERC Reference No. 2023-6900-23732).

## Abbreviations Used

ERC: Ethics Review Committee
WHO: World Health Organization
